# Integrated Analysis Reveals the Targets and Mechanisms in Immunosuppressive Effect of Mesalazine on Ulcerative Colitis

**DOI:** 10.3389/fnut.2022.867692

**Published:** 2022-05-19

**Authors:** Rong Li, Xue Huang, Lu Yang, Xiao Liang, Wenjun Huang, Keng Po Lai, Liming Zhou

**Affiliations:** ^1^Laboratory of Environmental Pollution and Integrative Omics, Guilin Medical University, Guilin, China; ^2^Department of Gastroenterology, Guigang City People's Hospital, The Eighth Affiliated Hospital of Guangxi Medical University, Guigang, China; ^3^Department of Pharmacology, West China School of Basic Medical Sciences and Forensic Medicine, Sichuan University, Chengdu, China

**Keywords:** ulcerative colitis, mesalazine, metabolomics, metagenomics, biomarkers

## Abstract

**Background:**

Ulcerative colitis (UC) is an inflammatory bowel disease that causes inflammation and ulcers in the digestive tract. Approximately 3 million US adults suffer from this disease. Mesalazine, an anti-inflammatory agent, is commonly used for the treatment of UC. However, some studies have demonstrated side effects of mesalazine, such as acute pancreatitis and hypereosinophilia. Therefore, a better understanding of the anti-inflammatory mechanism of mesalazine in UC could help improve the effectiveness of the drug and reduce its side effects. In this study, we used a dextran sodium sulfate-induced UC mouse model, and applied network pharmacology and omics bioinformatics approaches to uncover the potential pharmaceutical targets and the anti-inflammatory mechanism of mesalazine.

**Results:**

Network pharmacology analysis identified the core targets of mesalazine, biological processes, and cell signaling related to immunity and inflammatory responses mediated by mesalazine. Molecular docking analysis then indicated possible binding motifs on the core targets (including TNF-α, PTGS2, IL-1β, and EGFR). Metabolomics and 16S metagenomic analyses highlighted the correlation between gut microbiota and metabolite changes caused by mesalazine in the UC model.

**Conclusions:**

Collectively, the omics and bioinformatics approaches and the experimental data unveiled the detailed molecular mechanisms of mesalazine in UC treatment, functional regulation of the gut immune system, and reduction of intestinal inflammation. More importantly, the identified core targets could be targeted for the treatment of UC.

## Introduction

Inflammatory bowel diseases (IBD), mainly comprising ulcerative colitis (UC) and Crohn's disease, are complex multifactorial diseases (1). UC, a condition characterized by inflammation and ulceration of the colon and rectum, is a risk factor for colorectal cancer (2). The etiology of UC onset is complicated, and can be caused by complex synonym such as immune, genetic, environmental, and infection synonym (3). Epidemiological data indicate that due to changes in modern dietary habits, the incidence of UC has increased worldwide in recent years (4). According to the data from the Centers for Disease Control and Prevention in 2015, ~3 million US adults suffer from Crohn's disease or UC. More importantly, the number of diagnosed cases has increased significantly since 1999 (5). Recent studies have demonstrated that the occurrence and development of UC is closely related to disturbances in the intestinal flora. Intestinal bacteria play a regulatory role in maintaining gut homeostasis (6). Studies have shown that colonic bacteria in patients with UC can penetrate the mucus layer to interfere with epithelial cells and cause inflammation, suggesting that this could be the basis of the pathogenesis of UC (7). It has been reported that the chronic inflammatory response triggered by disturbance in gut microbiota homeostasis can cause infiltration of inflammatory cells, and the interaction between inflammatory factors can induce the development of UC (8). More importantly, there is accumulating evidence of a correlation between gut microbiota and host metabolite composition in different human diseases (9). Therefore, a better understanding of the pathogenic role of gut microbiota and metabolites in UC may reveal novel targets for tackling the disease and improving the efficiency of drug treatment.

Mesalazine (MZ), an active agent of 5-aminosalicylic acid, is prescribed for patients with UC because of its significant anti-inflammatory effect and safety (10). MZ inhibits the synthesis of inflammatory mediators and prostaglandins that cause inflammation and formation of leukotrienes (11). However, some clinical studies have demonstrated the side effects of MZ treatment, such as acute pancreatitis and hypereosinophilia (12, 13). Although some studies have explored the anti-UC molecular mechanism of MZ, further investigation in the form of mechanistic studies is needed to improve the effectiveness of MZ. The dextran sodium sulfate (DSS)-induced UC mouse model is commonly used to study UC pathogenesis. The model is simple to operate and acute or chronic UC can be easily induced in it by adjusting different intervention doses (14). Compared with other methods, the DSS-induced UC method has low cost, high success rate, and good reproducibility (15). The deterioration of intestinal tissue lesions, which is induced by DSS in mice with UC is similar to that in human patients with UC (16).

In this study, we first used network pharmacology and molecular docking to identify the core targets, biological functions, and therapeutic mechanisms of MZ. We used a DSS-induced UC mouse model, together with 16S ribosomal ribonucleic acid (16S rRNA) metagenomic sequencing and metabolomic analysis, to determine the structure and composition of gut microbiota in relation to the alteration of metabolite composition in UC. In addition, we aimed to determine the immunosuppressive effect of MZ through the modulation of the gut microbiota and metabolite composition. This study identified novel pharmaceutical targets of MZ. Moreover, we uncovered the relationship between the gut microbiota and metabolite composition in UC, to gain a better understanding of the molecular mechanism underlying the beneficial effect of MZ in UC treatment.

## Materials and Methods

### Identification of MZ- and UC-Associated Genes

MZ-associated genes were identified by searching in different databases, including Swiss Target Prediction, BATMAN TCM, and HitPick databases. Then, the UC-associated genes were extracted using Uniprot, GeneCard, OMIM, DisGeNET, and Therapeutic Target Database databases (17). Moreover, the identified genes of MZ and UC were compared to collect the human database-correlated targets using the online software Venn diagram (18).

### Interaction Network Analysis of the Common MZ- and UC-Associated Genes

The common MZ- and UC-associated genes were used to construct the gene network using STRING protein-protein interaction network functional enrichment analysis. Topological parameters from the median and maximum degrees of freedom were determined using the Network Analyzer of Cytoscape 3.7.1 software. The core targets were identified according to the degree value as follows: The median degree of freedom of the target point was 11.056, and the maximum degree of freedom was 40. Subsequently, the standard range of the core target was determined as 23–40 (19, 20). Core targets were subjected to gene ontology (GO) and Kyoto Encyclopedia of Genes Genomes (KEGG) pathway enrichment analysis. The terms and pathways were considered statistically significant if the *p*-value was < 0.05 (18, 21).

### Molecular Docking Testing

Molecular docking analysis was used to determine the possible binding of MZ to the core targets. The cytoarchitectures of the targeted proteins were obtained from the Protein Data Bank database. Then, ChemBio 3D software with ChemOffice setting was applied to the three-dimensional module of protein structures using molecular mechanics-2 analysis. Autodock Vina software was used to determine the functional crystal structures and molecular graphics, as previously described (22, 23).

### Animal Experimental Setup

Sixty C57BL/6 mice (6–7 weeks old, male) were purchased from Slack Jingda Experimental Animal Company (Changsha, Hunan) under the animal license number SCXK (Hunan) 2019-0004. All mouse experiments were approved by the Guilin Medical College-Experimental Animal Ethics Committee with an experimental animal ethics review number: GLMC202003117. The mice were kept in a relative humidity of 50% ± 10%, temperature of 20–25°C, and natural circadian rhythm light. Sixty mice were divided into three groups (20 mice each): (1) normal control group, (2) DSS group, and (3) DSS + MZ group. The normal control group freely drank pure water. On the other hand, the water was replaced with a 3% DSS solution in the DSS and DSS + MZ groups. The mice in the DSS + MZ group were given 0.8 g of MZ / body weight (kg) solution by gavage, once a day. The DSS-induced UC model was confirmed by the presence of bloody stool (Supplementary Figure 1). After the 7-day experimental period, the mice were euthanized by cervical dislocation, and the colorectal tissues were harvested and the feces were collected.

### Gut Metabolomics Analysis

The intestinal tissue was harvested for metabolomics analysis, the gut metabolites were extracted using protein precipitation with organic reagents, ground repeatedly with the metabolite extract, and placed in a refrigerated centrifuge at 4,000 g for 20 min. Chromatographic separation was performed using an ultra-high-performance liquid chromatography (UPLC) system (SCIEX, UK) ACQUITY UPLC T3 column (100 mm × 2.1 mm, 1.8 μm, Waters, UK), and TripleTOF 5600 high-resolution tandem mass spectrometer (SCIEX, UK) in positive and negative ion modes (PIM NIM) was used to determine the metabolites in the samples. The MS data collection adopted the IDA mode, and the TOF mass range was 60–1,200 Da. The original detection data were converted into readable data using the MSConvert software. The primary mass spectrometry information of metabolites was identified using the Human Metabolome Database (HMDB), while the KEGG database was used as a reference for annotation analysis. Secondary mass spectrometry information was matched with an in-house standard database. If the mass difference between the measured sample result and the database value was <10 ppm, an annotation was made. Then, the molecular formula of the metabolite was determined using isotope analysis. In addition, quantitative screening analysis of differential metabolites was performed to distinguish variables between groups of samples. The *t*-test was used to evaluate the difference in metabolite concentrations between the treatment and control groups. The screening conditions for metabolites were q < 0.05 and VIP ≥ 1.

### 16S RRNA Intestinal Metagenomic Sequencing

Fecal samples were collected using normal saline flushing enema, and mouse feces were collected by centrifugation and used for 16S rRNA sequencing, which was performed by the Lianchuan Biological Company (Hangzhou). Briefly, DNA was extracted from fecal samples of mice in each group using a stool DNA extraction kit, and primers were designed. The forward 341F (5 '- CCTACGGGNGGCWGCAG−3') and reverse 805R (5 '- GACTACHVGGGTATCTAATCC−3 ') were used for PCR amplification of the fecal 16S rRNA v3-v4 variable region. After amplification, the products were detected using gel electrophoresis and purified using AMPure XT beads. Finally, Qubit was used for DNA quantification. Purified samples were sequenced according to the manufacturer's instructions. The original data were filtered by fqtrim 0.94, merged with FLASH, and then filtered again using Vsearch software 2.3.4. The feature table and sequence of each group were demodulated by dada2, and the SILVA classifier was used to normalize the relative abundance of samples to the characteristic abundance. Bioinformatics analysis was performed using Qiime2 (19).

### Immunostaining

Colorectal samples were fixed with 4% paraformaldehyde buffer and prepared as 5 μm sections for immunohistochemical staining (24, 25). Briefly, the deparaffinized sections were incubated with 3% bovine serum albumin for 1 h. The sections were then incubated with 1:100 diluted primary antibodies against TNF-α, PTGS2, IL-1β, or EGFR (Bioss, Beijing, China) at 4°C overnight. After incubation with secondary antibodies, the antigen-antibody complex was stained with diaminobenzidine, and the nuclei were counterstained with hematoxylin (26, 27).

### Statistical Analysis

Statistical analysis of the data was conducted using GraphPad Prism 9 (GraphPad Software, San Diego, CA, USA). The data was tested for normal distribution using the Shapiro-Wilk test. Non-parametric statistical analysis between the multiple treatment groups was conducted using the Kruskal-Wallis test. *Post-hoc* pairwise comparisons between the groups were performed with the Dunn's test, to test statistical significance between the groups. The experimental data were expressed as the mean ± standard deviation. Significant results were determined using a cutoff of *p* < 0.05 and represented by asterisk.

## Results

### Mechanism of MZ-Mediated Anti-inflammatory Effect on UC

**Figure 1:**
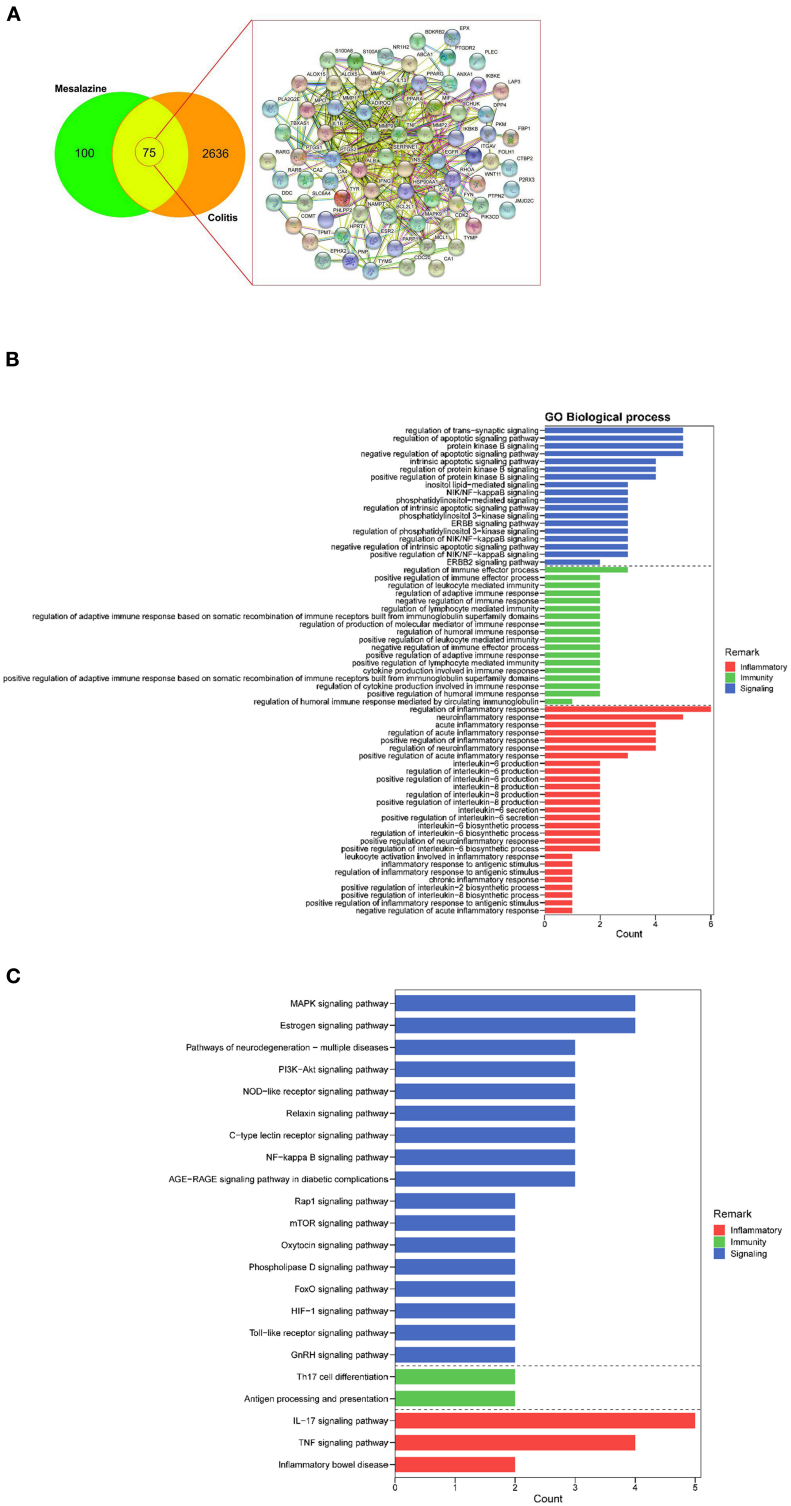
The identification of mesalazine (MZ)- and ulcerative colitis (UC)-associated genes and their immunosuppressive effect. **(A)** Venn diagrams show the number of common MZ- and colitis-associated genes. **(B)** The GO enrichment analysis highlighted the biological processes related to immunosuppressive effect of MZ on UC through the regulation of immunity, inflammatory responses, and cell signaling. **(C)** The KEGG pathway enrichment analysis highlighted the immunity and inflammatory response-related pathways controlled by MZ in DSS-induced UC model. The red bar presented inflammatory processes, the green bar represented the immunity, the blue bar represented the cell signaling pathways.

We identified 2711 UC-associated genes and 175 MZ-associated genes using a database search. Upon comparing these genes, 75 common targets were identified (Figure 1A). These common targets were subjected to STRING protein–protein interaction networks to determine the interaction between the common genes (Figure 1A). Nine core targets, namely ALB, TNF, INS, PTGS2, MMP9, IL-1β, EGFR, HSP90AA1, and MMP2, were identified using Cytoscape, that were associated with both MZ and UC (Supplementary Table 1). These nine core targets were subjected to GO and KEGG enrichment analyses. In the data analysis, we mainly focused on biological processes related to immunity, inflammatory responses, and cell signaling. Our results showed that the biological processes related to immunity consisted of regulation of immune effector processes, leukocyte-mediated immunity, lymphocyte-mediated immunity, humoral immune response, adaptive immune response based on somatic recombination of immune receptors built from immunoglobulin superfamily domains, and cytokine production involved in immune response (Figure 1B). In addition, we identified many biological processes related to inflammatory responses, such as regulation of acute and chronic inflammatory response, regulation of inflammatory response, regulation of neuroinflammatory response, interleukin-6 production and secretion, and biosynthetic processes of interleukin-2 and interleukin-8 (Figure 1B). In the KEGG pathway analysis, our results highlighted many cell signaling pathways related to cellular functions, namely MAPK signaling pathway, PI3K-Akt signaling pathway, TNF signaling pathway, relaxin signaling pathway, NOD-like receptor signaling pathway, mTOR signaling pathway, GnRH signaling pathway, oxytocin signaling pathway, phospholipase D signaling pathway, NF-kappa B signaling pathway, Toll-like receptor signaling pathway, HIF-1 signaling pathway, and FoxO signaling pathway (Figure 1C). More importantly, many pathways related to immunity (Th17 cell differentiation and antigen processing and presentation) and inflammatory responses (IL-17 signaling pathway, TNF signaling pathway, and IBD) were highlighted (Figure 1C). Taken together, our results suggest that MZ-mediated immunosuppressive effects are mediated by the regulation of different targets involved in immunity and inflammatory responses.

### MZ Inhibited the Induction of TNF, PTGS2, IL-1β, and EGFR in DSS-Induced UC Model

Molecular docking analysis was used to determine the possible binding of MZ to its target proteins, including TNF, PTGS2, IL-1β, and EGFR. For TNF (PDB ID: 6OOY), the free energy of binding to MZ was −4.3 kcal/mol through hydrogen bond formation with amino acid residues SER-60 (3.3 Å), LEU-120 (2.5 Å), and TYR-151 (2.9 Å) (Supplementary Figure 2A). For PTGS2 (PDB ID: 5IKR), the free energy of binding to MZ was −6.5 kcal/mol through hydrogen bond formation with TYR-385 (2.6 Å) and SER-530 (2.4 Å) (Supplementary Figure 2B). For IL-1β (PDB ID: 5R85), the free energy of binding to MZ was −5 kcal/mol through hydrogen bond formation with LEU-26 (3.0 Å) and LEU-82 (3.1 Å) (Supplementary Figure 2C). For EGFR (PDB ID: 5UGC), the free energy of binding to MZ was −6.1 kcal/mol through hydrogen bond formation withMET-793 (3.1 Å) (Supplementary Figure 2D). Then, we further investigated the effect of MZ on these proteins in DSS-induced UC model using immunostaining analysis. Our results showed elevated levels of TNF-α, PTGS2, IL-1β, and EGFR in the colorectal tissues of DSS-induced UC mice (Supplementary Figure 2E). And, treatment with MZ attenuated these inductions (Supplementary Figure 2E).

### Alteration of Gut Metabolites in DSS-Induced UC Model

**Figure 2:**
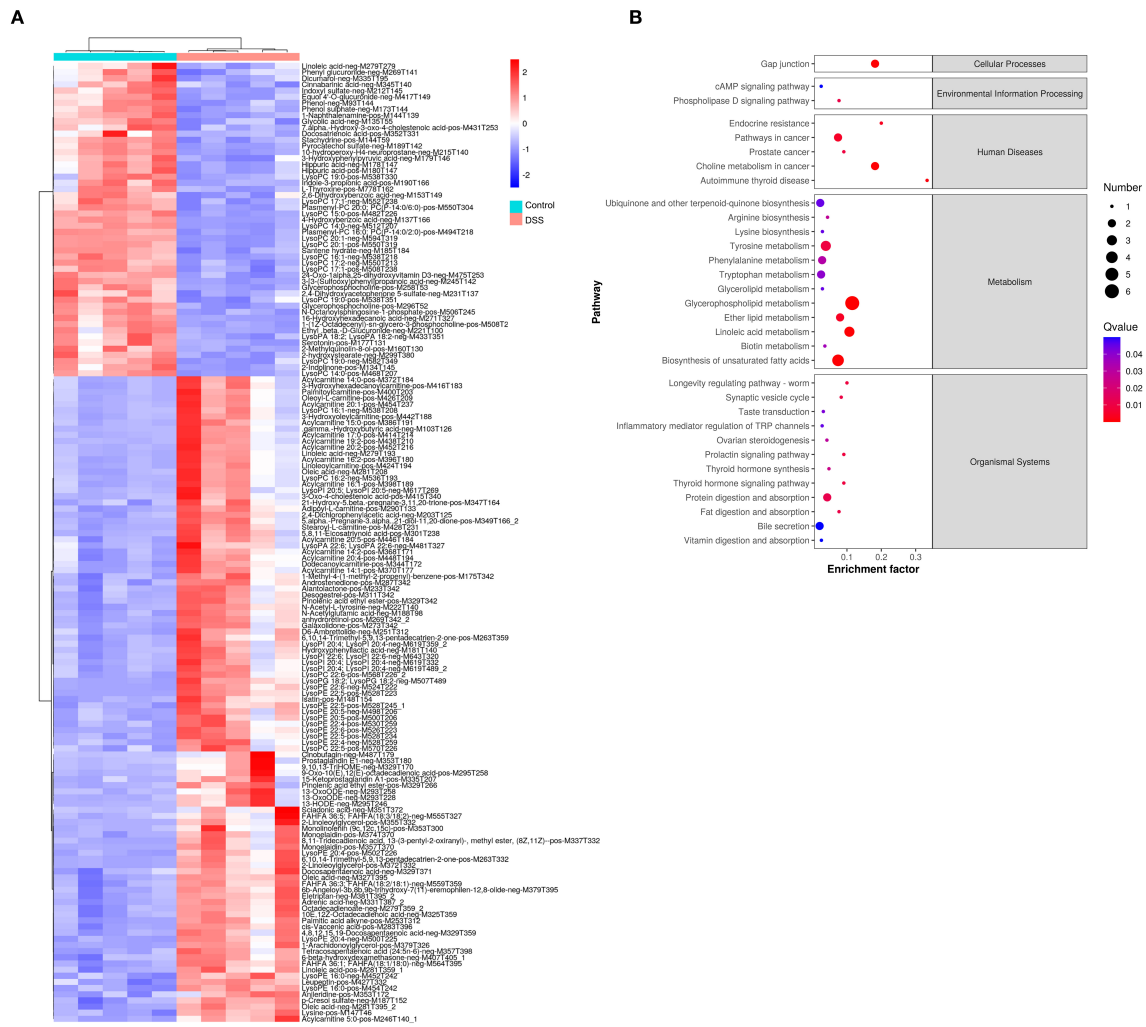
Change of gut metabolites in DSS-induced UC model. **(A)** Heatmap showed the level of gut metabolites in control group and DSS-induced colitis group. Red color represented the upregulation of metabolites. Blue color represented downregulated metabolites. **(B)** Rich factor plot showed the alteration of KEGG pathways in DSS-induced UC model. The size of dot represented the number of gene. The color intensity of dot represented the significance of the pathways.

Comparative metabolomic analysis was used to determine the metabolic changes in the DSS-induced UC model, and the effect of MZ on DSS-induced UC. Upon comparing the control group and the DSS-induced colitis group, we noted a significant dysregulation of 773 annotated metabolites, including 495 upregulated and 278 downregulated metabolites (Figure 2A), of which 156 metabolites were identified from a batch search on the human metabolome database (HMDB) in MS2 analysis. The dysregulated metabolites were subjected to KEGG enrichment analysis to understand the alteration of pathways in the DSS-induced UC model. Our results showed that DSS-induced UC caused significant dysregulation of a cluster of metabolites that are responsible for cellular processes, environmental information processing, human diseases, metabolism, and organismal systems (FDR <0.05) (Figure 2B). Autoimmune thyroid disease was highlighted in the category of human diseases. This was mainly caused by the downregulation of thyroxine. The inflammation mediated regulation of TRP channels was also altered in the DSS-induced UC model (Figure 2B). In addition, many metabolic pathways, including tyrosine metabolism, phenylalanine metabolism, tryptophan metabolism, arginine biosynthesis, lysine biosynthesis, glycerophospholipid metabolism, biosynthesis of unsaturated fatty acids, linoleic acid metabolism, ether lipid metabolism, glycerolipid metabolism, and biotin metabolism, were dysregulated in the DSS-induced UC model (Figure 2B).

### Anti-inflammatory Effect of MZ Is Mediated Through the Induction of Serotonin

**Figure 3:**
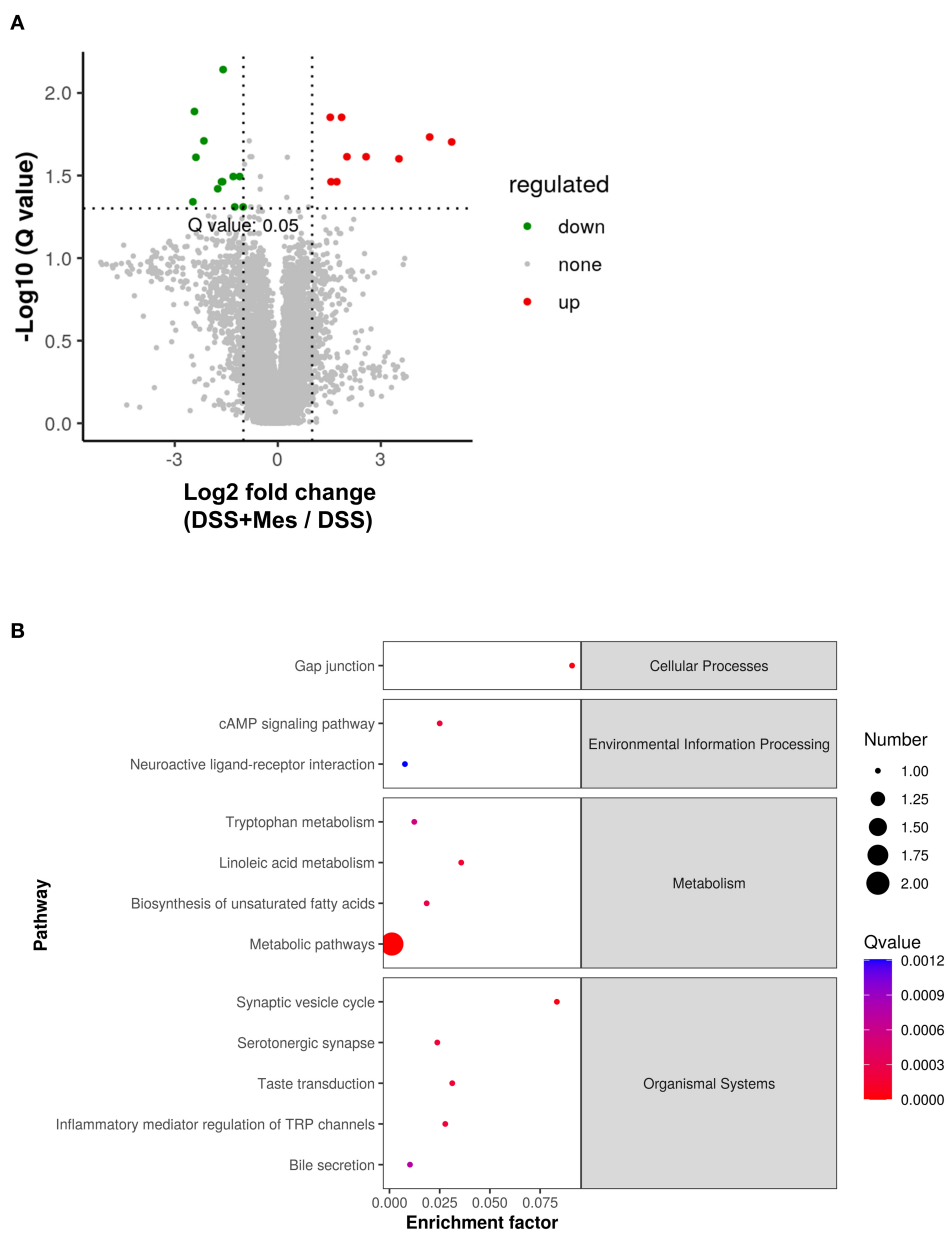
Alteration of gut metabolites in DSS-induced UC model caused by MZ treatment. **(A)** Volcano plot showed the change of gut metabolites after MZ treatment in DSS-induced UC model. Green dots represented reduced metabolites. Red dots represented induced metabolites. **(B)** Rich factor plot showed the alteration of KEGG pathways in DSS-induced UC model caused by MZ treatment. The size of dot represented the number of gene. The color intensity of dot represented the significance of the pathways.

To further understand the immunosuppressive effect of MZ on DSS-induced UC, we compared the metabolite changes in the DSS and DSS + MZ groups. We observed a significant change in 17 annotated metabolites, including 9 upregulated and 8 downregulated metabolites (Figure 3A). The KEGG pathway analysis of the dysregulated metabolites showed that the treatment with MZ altered many metabolic pathways, such as linoleic acid metabolism and biosynthesis of unsaturated fatty acids (Figure 3B). More importantly, the MZ treatment increased the levels of serotonin, leading to the mediation of tryptophan metabolism and inflammation mediated regulation of TRP channels (Figure 3B). In addition, we also observed the induction of paracetamol and linoleic acid by MZ treatment.

### Change in Gut Bacterial Community Richness in DSS-Induced UC Model Under MZ Treatment

Fecal 16S rRNA metagenomic analysis was used to determine the changes in the gut microbiome in the DSS-induced UC model after MZ treatment. At least 26 million valid bases were obtained for each sample (Supplementary Table 2). Operational taxonomic units (OTUs) were used to classify microbial diversity in terms of bacterial strains based on 16S rRNA gene sequence similarity. We identified 1,562 OTUs in the control group (Supplementary Figure 3A) and 1,452 OTUs in the DSS group (Supplementary Figure 3A), of which 398 OTUs were found to be common among the two groups (Supplementary Figure 3A). We then examined the effects of MZ on DSS-induced gut microbiome changes. We identified 1,340 OTUs in the DSS + MZ group (Supplementary Figure 3B), of which 430 OTUs were found to be common among the DSS and DSS + MZ groups (Supplementary Figure 3B). The identified OTUs were subjected to alpha diversity analysis to examine the species diversity in each sample using Shannon and Simpson indices, that reflect the richness and evenness of the bacterial community in the gut. When we compared the species diversity between the control, and DSS groups, we found a significant reduction in the Shannon (Supplementary Figure 3C) and Simpson (Supplementary Figure 3D) indices in the DSS group, as compared to those in the control group. More importantly, when we compared the species diversity between the DSS and DSS + MZ groups, we found that treatment with MZ could reverse the DSS-induced reduction in gut microbiome richness and evenness (Supplementary Figures 3C,D), reflected by the rebound of Shannon and Simpson indices in the DSS + MZ group as compared to those in the DSS group.

### Rebalancing of the Gut Microbial Taxa in DSS-Induced UC Model by MZ Treatment

We then investigated the changes in the taxonomic composition of each sample at different taxonomic levels. Our results showed that DSS-induced UC significantly reduced the number of gut microorganisms at the phylum level (Supplementary Figure 4A). The gut microbial species belonging to the phylum Patescibacteria were significantly reduced in the DSS group (Supplementary Figure 4A). This decrease was attributed mainly to the reduction in the abundance of the Saccharimonadia class (Supplementary Figure 4B). In addition, we observed a significant reduction in the classes Bacteroidia and Coriobacteriia (Supplementary Figure 4B). In contrast, an increase in the Bacilli and Verrucomicrobiae classes was observed in the DSS group (Supplementary Figure 4B). We further examined the effect of MZ on DSS altered gut bacterial taxonomic composition. We found that MZ treatment reversed the DSS-induced decrease in the orders Betaproteobacteriales and Coriobacteriales (Table 1). At the genus level, we observed a DSS-induced reduction in *Anaerotignum, Enterorhabdus, Erysipelatoclostridium*, and *Lachnospiraceae_UCG-006*. Interestingly, treatment with MZ reversed these reductions (Table 2). The dysregulated gut microbiota was subjected to phylogenetic investigation of communities using reconstruction of unobserved states (PICRUSt), an ancestral state reconstruction algorithm. The results of KEGG orthology (KO) showed 73 significant alterations of pathways (*p* < 0.05), including the activation of L-histidine degradation, I myo-, chiro-scillo-inositol degradation, NAD biosynthesis II (from tryptophan), and L-tryptophan degradation to 2-amino-3-carboxymuconate semialdehyde (Supplementary Figure 4C), caused by MZ treatment. In addition, we observed the downregulation of enterobactin biosynthesis, fucose degradation, and a group of super pathways, including the biosynthesis of (Kdo)2-lipid A L-tryptophan, as well as the degradation of L-tryptophan, L-arginine, L-ornithine, L-arginine, putrescine, and 4-aminobutanoate (Supplementary Figure 4C).

**Table 1:** MZ treatment reversed the DSS-altered gut bacterial taxonomic composition at order level.

**Order**	**log2 fold change (DSS/Ctrl)**	**wilcoxt test p-value**	**regulation (DSS/Ctrl)**	**log2 fold change (DSS+mesalazine/DSS)**	**wilcoxt test p-value**	**regulation (DSS+mesalazine/DSS)**
Anaeroplasmatales	2.89	0.03	up	−0.68	0.35	down
Bacteroidales	−1.44	0.01	down	0.13	0.75	up
Betaproteobacteriales	−2.48	0.01	down	2.38	0.05	up
Coriobacteriales	−3.78	0.01	down	2.44	0.03	up
Enterobacteriales	6.60	0.02	up	−3.54	0.08	down
Firmicutes	3.15	0.01	up	−0.15	0.92	down
Lactobacillales	2.19	0.02	up	3.29	0.17	up
Saccharimonadales	−1.86	0.05	down	0.30	0.46	up
Verrucomicrobiales	9.63	0.01	up	−0.29	0.25	down

**Table 2:** MZ treatment reversed the DSS-altered gut bacterial taxonomic composition at genus level.

**Genus**	**log2 fold change (DSS/Ctrl)**	**wilcoxt test p-value**	**regulation (DSS/Ctrl)**	**log2 fold change (DSS+mesalazine/DSS)**	**wilcoxt test p-value**	**regulation (DSS+mesalazine/DSS)**
g__Anaerotignum	−1.96	0.009	down	1.01	0.028	up
g__Burkholderia-Caballeronia- Paraburkholderia	−2.53	0.009	down	2.76	0.047	up
g__Enterorhabdus	−3.94	0.009	down	2.58	0.016	up
g__Erysipelatoclostridium	−2.10	0.009	down	2.28	0.009	up
g__Lachnospiraceae_UCG-006	−2.38	0.016	down	2.35	0.047	up

## Discussion

UC is a common chronic inflammatory gastrointestinal disease that affects gut function (28). While the pharmacological treatment options of UC are limited, MZ has been reported to be safe and effective for this purpose (10, 29). In patients with UC, MZ was found to inhibit the transcription factor NF-kappa B in inflamed mucosa in biopsies (30). However, many reports have demonstrated the potential side effects of MZ. For instance, MZ was reported to induce acute pancreatitis and eosinophilic glossitis in patients with UC (12, 13). Therefore, a better understanding of the molecular targets and mechanisms underlying the effect of MZ would help reduce its side effects and improve its effectiveness in treating UC. In this study, we used omics and bioinformatic analyses to delineate the biological functions and signaling pathways affected by MZ treatment.

First, the network pharmacology analysis identified the core targets of MZ, including ALB, TNF, INS, PTGS2, MMP9, IL-1β, EGFR, HSP90AA1, and MMP2. The GO and KEGG enrichment analyses further highlighted the immunosuppressive effect of MZ through the regulation of immunity, inflammation, and various cell signaling pathways. One of the important pathways affected by MZ is TNF signaling. TNF, also known as TNF-α, is a well-known proinflammatory cytokine that is released by white blood cells and can induce systemic or cellular inflammation, causing rheumatoid arthritis (31). Thus, the inhibition of TNF-α is an effective strategy for treating UC, as reported in clinical and animal studies (32, 33). Furthermore, molecular docking also indicated the targeting of TNF by MZ. In addition, our results highlighted the binding of MZ to PTGS2, IL-1β, and EGFR. PTGS2, alternatively termed COX-2, is an inflammatory enzyme associated with intestinal inflammatory disease like IBD (34). A previous clinical study has demonstrated a negative correlation between the protein levels of COX-2 and the disease severity of IBD (35). A review has also suggested that COX-2 can cause the clinical manifestations of a bacterial infection, including inflammation and septic shock (36). Moreover, the safety and efficacy of COX-2 inhibitors in patients with IBD have been previously reported in clinical practice (37). IL-1β is a pro-inflammatory cytokine that plays an essential role in acute or chronic inflammatory responses (38). A single nucleotide polymorphism study on IDB patients showed that a mutant of IL-1β increased the proportion of IBD-associated colorectal cancer in the population (39). More importantly, an increase in IL-1β levels was found to be associated with increased severity of IBD (40). A study involving DSS-induced UC in mice demonstrated the induction of IL-1β through NLRP3 inflammasome activation (41). EGFR, a 170 kDa transmembrane tyrosine kinase, can activate target genes in the nucleus to promote cell division and proliferation (42). In a preclinical study, an EGFR-dependent mechanism of intestinal injury was identified in DSS-induced UC mice (43). More importantly, these targets were found to be induced in our DSS-induced UC mouse model as well, and the treatment with MZ could reverse their induction, suggesting that all these targets can be used to evaluate the immunosuppressive effect of MZ. In addition, they can be used as novel markers for possible combination therapy for treating UC.

In the second part of the study, we used comparative metabolomic analysis to understand the role of gut metabolism modulation in the immunosuppressive effect of MZ on DSS-induced UC. We identified many metabolic dysregulations in the DSS-induced UC model. Most of these metabolic pathways play a pathological role in UC. For instance, tyrosine metabolism controls the various ways in which tyrosine is catabolized or transformed to generate a wide variety of biologically important molecules. Tyrosine kinases catalyze the phosphorylation of tyrosine residues in proteins. It has been reported that tyrosine kinase 2 (TYK2) plays an important role in inflammation through pro- and anti-inflammatory cytokines, and is involved in the pathogenesis of IBD (44, 45). Our results also highlighted the alteration of tryptophan metabolism in the DSS-induced UC model. Tryptophan metabolism is also associated with IBD (46). Moreover, tryptophan is a gut microbiota-derived metabolite that regulates inflammation in UC (47). In addition, inflammation induces tryptophan metabolism through the kynurenine pathway and yields immunologically relevant metabolites in UC, suggesting a close association between tryptophan metabolism and UC (48). In addition, we found a significant downregulation of thyroxine in the DSS-induced UC model. Thyroxine, also known as T4, plays a crucial role in digestive function, metabolism, and muscle control. In the digestive system, thyroxine increases the secretion of digestive juices and promotes smooth muscle function, thus facilitating contractions of gastric motility (49). Therefore, the reduction in thyroxine levels leads to malfunction of the digestive system.

To delineate the molecular mechanism underlying the beneficial effects of MZ in UC treatment, we used metabolomic analysis to determine the metabolite changes and their functional implications in MZ treatment. Although we only observed a small number of metabolite changes under the treatment, the pathway analysis highlighted that the changes in metabolites were related to the alteration of linoleic acid metabolism and biosynthesis of unsaturated fatty acids. Linoleic acid, a polyunsaturated omega-6 fatty acid, is commonly found in red meat and many oils. A cohort study conducted in Europe reported that a high dietary intake of linoleic acid increased the risk of developing incident UC (50). Another animal study also demonstrated that partial replacement of dietary linoleic acid with long-chain n-3 polyunsaturated fatty acids protected against DSS-induced UC in rats (51), indicating a role of linoleic acid in the pathogenesis of UC. In our study, we also observed the modulation of unsaturated fatty acids by MZ treatment. It has been reported that an imbalance in polyunsaturated fatty acids is one of the causes of IBD (52), because the modification of fatty acid metabolism stimulates inflammatory cytokines, leading to inflammation and UC (53). Therefore, MZ treatment could restore the levels of polyunsaturated fatty acids. In addition, it also controlled tryptophan metabolism and inflammation mediated regulation of TRP channels. The impact of gut microbiota on the intestinal immunity mediated by tryptophan metabolism has been previously reported (54). Tryptophan plays a crucial role in maintaining the balance between intestinal immune tolerance and preservation of the gut microbiota, which in turn influences gut immune homeostasis and the intestinal immune response (55). Our data showed that most of the effects of MZ treatment were achieved through the induction of serotonin. Serotonin plays a key role in the modulation of chronic inflammatory processes, including autoimmune diseases, through its immunomodulatory effect on the interaction between the nervous and immune systems (56). In addition, serotonin receptors regulate inflammatory responses in experimental colitis (57). A mouse study demonstrated that prophylactic administration of tryptophan could ameliorate colitis through its impact on serotonin receptor signaling (58). Our findings suggest that MZ treatment increases the levels of serotonin to mediate different signaling pathways, to ameliorate the autoimmune activity in UC.

UC is a systemic disease characterized by immunological alterations in the colon. Gut microbiome performs pivotal functions like promotion of digestion, xenobiotic metabolism, and regulation of innate and adaptive immunological processes (59). Many studies have demonstrated a close association between gut microbiota and gut metabolites (60). It has been reported that gut microbes play important roles in gastrointestinal health and disease, ranging from protective to pro-inflammatory actions (61). In context of regulatory T cells, it is plausible that the gut microbiome may play a role in other chronic immune-mediated inflammatory diseases. Therefore, in the last part of the study, we used 16S metagenomic sequencing to determine the changes in the gut microbiota in response to DSS-induced UC, aiming to understand the role of alteration of gut microbiota in the anti-inflammatory effect of MZ on UC. In our study, we found a significant reduction in the richness and evenness of the gut microbiome diversity in DSS-induced UC. This finding is similar to that of a recent study, in which patients with IBD were found to harbor, on average, 25% fewer microbial genes than healthy people (62). More importantly, a study of the gut of obese Danish individuals indicated that gut samples with low bacterial richness are characterized by a more pronounced inflammatory phenotype (63). This response could be explained by changes in host metabolite composition related to inflammation (64). Furthermore, we found that MZ reversed the reduction in richness and evenness of the gut microbiome in the DSS-induced UC model, suggesting a positive effect of the treatment. Interestingly, in context of the alterations in bacterial taxonomy, we observed that MZ treatment could reverse the colitis-induced reduction in the order Coriobacteriales. Bacteria of this order are gram-positive and belong to the phylum Actinobacteria. Moreover, it has been reported that the order Coriobacteriales increases significantly in the ceca of mice in response to stress (65) and is associated with changes in the inflammation phenotype (66). In addition, we observed a similar change in bacterial taxonomy following treatment with MZ. It includes the rebalancing of *Enterorhabdus*, which is a gram-positive and non-spore-forming bacillus that exists in mammalian intestines, and is associated with inflamed gut mucosa. It has been reported that the levels of *Enterorhabdus* are associated with Toll-like receptors (TLRs), which play a valuable role in the intestinal mucosal immune system by mediating TLR4/MyD88 signal transduction (67). Additionally, an increase in *Enterorhabdus* was reported to improve intestinal barrier integrity in the gut of a non-alcoholic fatty liver disease model (68). MZ treatment reversed the reduction in *Erysipelatoclostridium* and *Lachnospiraceae UCG-006*, indicating high efficacy of MZ in the treatment of UC. A DSS-induced UC mouse study demonstrated the association of *Erysipelatoclostridium* with reduced inflammatory intestinal damage (69). *Lachnospiraceae UCG-006* has been reported to regulate intestinal homeostasis and physiology (70). More importantly*, Lachnospiraceae UCG-006* was found to regulate the immune system and gut microbiota through its antiallergic and anti-inflammatory effects (71). Lastly, we used PICRUSt to determine the role of the rebalancing of bacterial taxonomy by MZ treatment. Our data demonstrated that MZ treatment could lead to a reduction in enterobactin biosynthesis. Enterobactin, a catecholate siderophore, is a potent inhibitor of myeloperoxidase. It suppresses the host innate immune response during inflammatory gut diseases (72). The reduced bioactivity of enterobactin was reported to enhance the tolerance to adverse pH, high concentrations of bile acids, and oxidative stress in the inflamed gut (73), leading to protection against intestinal inflammation (74). In addition, we found that MZ treatment might increase fructose levels by suppressing fructose degradation. It has been reported that fructose ameliorates DSS-induced acute UC by inhibiting M1 macrophage polarization as well as the NLRP3 inflammasome (75). Moreover, fucose protects the gut from intestinal inflammation by modulating the crosstalk between bile acids and gut microbiota in a chronic UC model (76).

In conclusion, the identified molecular targets and gut microbial taxa with differential abundance patterns common in UC may serve as biomarkers for the detection of UC, and indicate that there may be a common component of colitis etiology. Our work sheds light on the impact of MZ on the gut microbiome and innate immune responses in context of the treatment of UC. A better understanding of the mechanism of action of MZ could help identify optional targets for combined therapy, leading to increase in effectiveness and reduction of the side effects of MZ in UC treatment.

## Data Availability Statement

The datasets presented in this study can be found in online repositories. The names of the repository/repositories and accession number(s) can be found below: https://www.ncbi.nlm.nih.gov/, PRJNA827915.

## Ethics Statement

The animal study was reviewed and approved by Guilin Medical University.

## Author Contributions

RL, KL, and LZ: conceptualization. RL, XH, LY, XL, and WH: data curation. KL: funding acquisition. RL: investigation. RL, LY, and XL: methodology. LY and XL: software. LZ: supervision. WH, KL, and LZ: roles/writing—original draft. All authors contributed to the article and approved the submitted version.

## Conflict of Interest

The authors declare that the research was conducted in the absence of any commercial or financial relationships that could be construed as a potential conflict of interest.

## Publisher's Note

All claims expressed in this article are solely those of the authors and do not necessarily represent those of their affiliated organizations, or those of the publisher, the editors and the reviewers. Any product that may be evaluated in this article, or claim that may be made by its manufacturer, is not guaranteed or endorsed by the publisher.

## Supplementary Material

Supplementary Figure 1 The pictures of stool samples collected from control, DSS-induced colitis group and DSS-induced colitis + MZ group.

Supplementary Figure 2 MZ targeted relieved the elevation of TNF-α, PTGS2, IL-1β, and EGFR in DSS-induced UC model. **(A)** The binding of MZ with TNF. **(B)** The binding of MZ with PTGS2. **(C)** The binding of MZ with IL-1β **(D)** The binding of MZ with EGFR. **(E)** Immunostaining analysis demonstrated that MZ treatment markedly suppressed the induced expressions of TNF-α, PTGS2, IL-1β, and EGFR in colorectal of DSS-induced UC model. The experimental data were expressed as the mean ± standard deviation. *Post-hoc* pairwise comparisons between the groups were performed with the Dunn's test, to test statistical significance between the groups. The experimental data were expressed as the mean ± standard deviation. Significant results were determined using a cutoff of *p* < 0.05 and represented by asterisk.

Supplementary Figure 3 Relief of the DSS-altered gut microbiota by MZ treatment. **(A)** Venn diagram showed the operational taxonomic unit (OTU) shared by control group and DSS group. **(B)** Venn diagram showed the OTU shared by DSS group and DSS + MZ group. **(C)** Shannon index and **(D)** Simpson index showed the relieve of bacterial community's richness evenness caused by MZ treatment. The data were expressed as the mean ± standard deviation. *Post-hoc* pairwise comparisons between the groups were performed with the Dunn's test, to test statistical significance between the groups. The experimental data were expressed as the mean ± standard deviation. Significant results were determined using a cutoff of *p* < 0.05 and represented by asterisk.

Supplementary Figure 4 The change of gut microbiome taxonomy in DSS-induced UC model. **(A)** DSS induced gut microbial community change in phylum level. **(B)** DSS-induced gut microbial community change in class level. **(C)** Prediction of functional change in DSS-induced UC model caused by MZ treatment. The experimental data were expressed as the mean ± standard deviation. *Post-hoc* pairwise comparisons between the groups were performed with the Dunn's test, to test statistical significance between the groups. The experimental data were expressed as the mean ± standard deviation. Significant results were determined using a cutoff of *p* < 0.05 and represented by asterisk.

## Abbreviations

UC: ulcerative colitis
IBD: inflammatory bowel diseases
MZ: Mesalazine
DSS: dextran sodium sulfate
16S rRNA: 16S ribosomal ribonucleic acid
GO: gene ontology
KEGG: Kyoto Encyclopedia of Genes Genomes
UPLC: ultra-high-performance liquid chromatography
HMDB: Metabolome Database
PIM: positive ion mode
NIM: negative ion mode.

## References

[1] Chassaing B Aitken JD Malleshappa M Vijay-Kumar M. Dextran sulfate sodium (DSS)-induced colitis in mice. Curr Protoc Immunol. (2014) 104:15.25.1–14. 10.1002/0471142735.im1525s104 24510619 PMC3980572

[2] Rogler G. Chronic ulcerative colitis and colorectal cancer. Cancer Lett. (2014) 345:235–241. 10.1016/j.canlet.2013.07.032 23941831

[3] Ungaro R Mehandru S Allen PB Peyrin-Biroulet L Colombel JF. Ulcerative colitis. Lancet. (2017) 389:1756–70. 10.1016/S0140-6736(16)32126-2 27914657 PMC6487890

[4] Ananthakrishnan AN. Epidemiology and risk factors for IBD. Nat Rev Gastroenterol Hepatol. (2015) 12:205–17. 10.1038/nrgastro.2015.34 25732745

[5] Kucharzik T Koletzko S Kannengiesser K Dignass A. Ulcerative colitis-diagnostic and therapeutic algorithms. Dtsch Arztebl Int. (2020) 117:564–74. 10.3238/arztebl.2020.0564 33148393 PMC8171548

[6] Guo XY Liu XJ Hao JY. Gut microbiota in ulcerative colitis: insights on pathogenesis and treatment. J Dig Dis. (2020) 21:147–59. 10.1111/1751-2980.12849 32040250

[7] Nishida A Inoue R Inatomi O Bamba S Naito Y Andoh A. Gut microbiota in the pathogenesis of inflammatory bowel disease. Clin J Gastroenterol. (2018) 11:1–10. 10.1007/s12328-017-0813-5 29285689

[8] Larabi A Barnich N Nguyen HTT. New insights into the interplay between autophagy, gut microbiota and inflammatory responses in IBD. Autophagy. (2020) 16:38–51. 10.1080/15548627.2019.1635384 31286804 PMC6984609

[9] Li R Huang X Liang X Su M Lai KP Chen J. Integrated omics analysis reveals the alteration of gut microbe-metabolites in obese adults. Brief Bioinform. (2021) 22:bbaa165. 10.1093/bib/bbaa165 32770198

[10] Sehgal P Colombel JF Aboubakr A Narula N. Systematic review: safety of mesalazine in ulcerative colitis. Aliment Pharmacol Ther. (2018) 47:1597–609. 10.1111/apt.14688 29722441

[11] Lauritsen K Laursen LS Bukhave K Rask-Madsen J. Effects of topical 5-aminosalicylic acid and prednisolone on prostaglandin E2 and leukotriene B4 levels determined by equilibrium *in vivo* dialysis of rectum in relapsing ulcerative colitis. Gastroenterology. (1986) 91:837–44. 10.1016/0016-5085(86)90684-0 3017804

[12] Correia JP Ponte AI Silva JC Gomes AC Afecto E. Mesalazine-induced acute pancreatitis: a rare adverse reaction but with important therapeutic implications in ulcerative colitis. Eur J Gastroenterol Hepatol. (2021) 33:595. 10.1097/MEG.0000000000001901 33657605

[13] Smets G Grosber M Gutermuth J Bravenboer B Velkeniers B. Mesalazine-induced eosinophilic glossitis and hypereosinophilia in a patient with ulcerative colitis: a case report and review of literature. J Eur Acad Dermatol Venereol. (2021) 35:e462–4. 10.1111/jdv.17223 33725368

[14] Eichele DD Kharbanda KK. Dextran sodium sulfate colitis murine model: an indispensable tool for advancing our understanding of inflammatory bowel diseases pathogenesis. World J Gastroenterol. (2017) 23:6016–29. 10.3748/wjg.v23.i33.6016 28970718 PMC5597494

[15] Jialing L Yangyang G Jing Z Xiaoyi T Ping W Liwei S. Changes in serum inflammatory cytokine levels and intestinal flora in a self-healing dextran sodium sulfate-induced ulcerative colitis murine model. Life Sci. (2020) 263:118587. 10.1016/j.lfs.2020.118587 33065145

[16] Wirtz S Neufert C Weigmann B Neurath MF. Chemically induced mouse models of intestinal inflammation. Nat Protoc. (2007) 2:541–6. 10.1038/nprot.2007.41 17406617

[17] Li R Guo C Li Y Qin Z Huang W. Therapeutic targets and signaling mechanisms of vitamin c activity against sepsis: a bioinformatics study. Brief Bioinform. (2020) 22:bbaa079. 10.1093/bib/bbaa079 32393985 PMC7454291

[18] Li R Li Y Liang X Yang L Su M Lai KP. Network Pharmacology and bioinformatics analyses identify intersection genes of niacin and COVID-19 as potential therapeutic targets. Brief Bioinform. (2020) 22:1279–90. 10.1093/bib/bbaa300 33169132 PMC7717147

[19] Zhou R Wu K Su M Li R. Bioinformatic and experimental data decipher the pharmacological targets and mechanisms of plumbagin against hepatocellular carcinoma. Environ Toxicol Pharmacol. (2019) 70:103200. 10.1016/j.etap.2019.103200 31158732

[20] Wu K Wei P Liu M Liang X Su M. To reveal pharmacological targets and molecular mechanisms of curcumol against interstitial cystitis. J Adv Res. (2019) 20:43–50. 10.1016/j.jare.2019.05.003 31193808 PMC6543129

[21] Li R Guo C Li Y Liang X Yang L Huang W. Therapeutic target and molecular mechanism of vitamin C-treated pneumonia: a systematic study of network pharmacology. Food Funct. (2020) 11:4765–72. 10.1039/D0FO00421A 32420559

[22] Li R Guo C Li Y Liang X Su M. Functional benefit and molecular mechanism of vitamin C against perfluorooctanesulfonate-associated leukemia. Chemosphere. (2021) 263:128242. 10.1016/j.chemosphere.2020.128242 33297189

[23] Nong Y Liang Y Liang X Li Y Yang B. Pharmacological targets and mechanisms of calycosin against meningitis. Aging. (2020) 1:19468–92. 10.18632/aging.103886 33031061 PMC7732281

[24] Xu X Guo C Liang X Li R Chen J. Potential biomarker of fibroblast growth factor 21 in valproic acid-treated livers. Biofactors. (2019) 45:740–749. 10.1002/biof.1519 31120577

[25] Huang W Su L Zhang X Xu X Li R. Endocrinological characterization of pancreatic ducts in HFD and HGD fed mice. J Cell Biochem. (2019) 120:16153–59. 10.1002/jcb.28896 31081956

[26] Zhou R Liu M Liang X Su M Li R. Clinical features of aflatoxin B1-exposed patients with liver cancer and the molecular mechanism of aflatoxin B1 on liver cancer cells. Environ Toxicol Pharmacol. (2019) 71:103225. 10.1016/j.etap.2019.103225 31376682

[27] Zhou R Xu X Liu M Wu X Li R. Immunophenotypes of ductal epithelial cells in advanced pancreatic ductal adenocarcinoma. Digestion. (2019) 99:247–51. 10.1159/000492861 30205390

[28] Mourad FH Barada KA Saade NE. Impairment of small intestinal function in ulcerative colitis: role of enteric innervation. J Crohns Colitis. (2017) 11:369–77. 10.1093/ecco-jcc/jjw162 27655154

[29] Criscuoli V Modesto I Orlando A Cottone M. Mesalazine for the treatment of inflammatory bowel disease. Expert Opin Pharmacother. (2013) 14:1669–78. 10.1517/14656566.2013.808622 23767798

[30] Bantel H Berg C Vieth M Stolte M Kruis W Schulze-Osthoff K. Mesalazine inhibits activation of transcription factor NF-kappaB in inflamed mucosa of patients with ulcerative colitis. Am J Gastroenterol. (2000) 95:3452–7. 10.1111/j.1572-0241.2000.03360.x 11151876

[31] Vashisht P O'dell J. Not all TNF inhibitors in rheumatoid arthritis are created equal: important clinical differences. Expert Opin Biol Ther. (2017) 17:989–99. 10.1080/14712598.2017.1340453 28594252

[32] Taxonera C Rodríguez C Bertoletti F Menchén L Arribas J Sierra M. Clinical outcomes of golimumab as first, second or third anti-TNF agent in patients with moderate-to-severe ulcerative colitis. Inflamm Bowel Dis. (2017) 23:1394–402. 10.1097/MIB.0000000000001144 28671873

[33] Xiao YT Yan WH Cao Y Yan JK Cai W. Neutralization of IL-6 and TNF-α ameliorates intestinal permeability in DSS-induced colitis. Cytokine. (2016) 83:189–92. 10.1016/j.cyto.2016.04.012 27155817

[34] Speciale A Muscarà C Molonia MS Toscano G Cimino F Saija A. *In vitro* protective effects of a standardized extract from *Cynara cardunculus* L. leaves against TNF-α-induced intestinal inflammation. Front Pharmacol. (2022) 13:809938. 10.3389/fphar.2022.809938 35222027 PMC8874283

[35] Gao L Yu Q Zhang H Wang Z Zhang T Xiang J. A resident stromal cell population actively restrains innate immune response in the propagation phase of colitis pathogenesis in mice. Sci Transl Med. (2021) 13:eabb5071. 10.1126/scitranslmed.abb5071 34290057

[36] Smith WL DeWitt DL Garavito RM. Cyclooxygenases: structural, cellular, and molecular biology. Annu Rev Biochem. (2000) 69:145–82. 10.1146/annurev.biochem.69.1.145 10966456

[37] Mahadevan U Loftus EV Jr Tremaine WJ Sandborn WJ. Safety of selective cyclooxygenase-2 inhibitors in inflammatory bowel disease. Am J Gastroenterol. (2002) 97:910–14. 10.1111/j.1572-0241.2002.05608.x 12008668

[38] Dinarello CA. Interleukin-1 in the pathogenesis and treatment of inflammatory diseases. Blood. (2011) 117:3720–32. 10.1182/blood-2010-07-273417 21304099 PMC3083294

[39] Li H Jin Z Li X Wu L Jin J. Associations between single-nucleotide polymorphisms and inflammatory bowel disease-associated colorectal cancers in inflammatory bowel disease patients: a meta-analysis. Clin Transl Oncol. (2017) 19:1018–27. 10.1007/s12094-017-1634-1 28243990

[40] Coccia M Harrison OJ Schiering C Asquith MJ Becher B Powrie F. IL-1β mediates chronic intestinal inflammation by promoting the accumulation of IL-17A secreting innate lymphoid cells and CD4(+) Th17 cells. J Exp Med. (2012) 209:1595–609. 10.1084/jem.20111453 22891275 PMC3428945

[41] Gong Z Zhao S Zhou J Yan J Wang L Du X. Curcumin alleviates DSS-induced colitis via inhibiting NLRP3 inflammsome activation and IL-1β production. Mol Immunol. (2018) 104:11–19. 10.1016/j.molimm.2018.09.004 30396035

[42] Lui VW Grandis JR. EGFR-mediated cell cycle regulation. Anticancer Res. (2002) 22:1–11. 12017269

[43] Yan F Cao H Cover TL Washington MK Shi Y Liu L. Colon-specific delivery of a probiotic-derived soluble protein ameliorates intestinal inflammation in mice through an EGFR-dependent mechanism. J Clin Invest. (2011) 121:2242–53. 10.1172/JCI44031 21606592 PMC3104743

[44] Page TH Smolinska M Gillespie J Urbaniak AM Foxwell BM. Tyrosine kinases and inflammatory signalling. Curr Mol Med. (2009) 9:69–85. 10.2174/156652409787314507 19199943

[45] De Vries LCS Ghiboub M van Hamersveld PHP Welting O Verseijden C Bell MJ. Tyrosine kinase 2 signalling drives pathogenic T cells in colitis. J Crohns Colitis. (2021) 15:617–30. 10.1093/ecco-jcc/jjaa199 33005945 PMC8023831

[46] Nikolaus S Schulte B Al-Massad N Thieme F Schulte DM Bethge J. Increased tryptophan metabolism is associated with activity of inflammatory bowel diseases. Gastroenterology. (2017) 153:1504–16.e2. 10.1053/j.gastro.2017.08.028 28827067

[47] Etienne-Mesmin L Chassaing B Gewirtz AT. Tryptophan: a gut microbiota-derived metabolites regulating inflammation. World J Gastrointest Pharmacol Ther. (2017) 8:7–9. 10.4292/wjgpt.v8.i1.7 28217370 PMC5292609

[48] Sofia MA Ciorba MA Meckel K Lim CK Guillemin GJ Weber CR. Tryptophan metabolism through the kynurenine pathway is associated with endoscopic inflammation in ulcerative colitis. Inflamm Bowel Dis. (2018) 24:1471–80. 10.1093/ibd/izy103 29796641 PMC6196764

[49] Bhattacharyya SK Chaki KK Misra KK. Effect of thyroxine on some digestive enzymes of the adult male toad, Bufo melanostictus. Folia Biol. (2002) 50:83–90. 12597539

[50] IBD in EPIC Study Investigators Tjonneland A Overvad K Bergmann MM Nagel G Linseisen J. Linoleic acid, a dietary n-6 polyunsaturated fatty acid, and the aetiology of ulcerative colitis: a nested case-control study within a European prospective cohort study. Gut. (2009) 58:1606–11. 10.1136/gut.2008.169078 19628674

[51] Tyagi A Kumar U Santosh VS Reddy S Mohammed SB Ibrahim A. Partial r eplacement of dietary linoleic acid with long chain n-3 polyunsaturated fatty acids protects against dextran sulfate sodium-induced colitis in rats. Prostaglandins Leukot Essent Fatty Acids. (2014) 91:289–97. 10.1016/j.plefa.2014.09.003 25451558

[52] Scaioli E Liverani E Belluzzi A. The imbalance between n-6/n-3 polyunsaturated fatty acids and inflammatory bowel disease: a comprehensive review and future therapeutic perspectives. Int J Mol Sci. (2017) 18:2619. 10.3390/ijms18122619 29206211 PMC5751222

[53] Wiese DM Horst SN Brown CT Allaman MM Hodges ME Slaughter JC. Serum fatty acids are correlated with inflammatory cytokines in ulcerative colitis. PLoS ONE. (2016) 11:e0156387. 10.1371/journal.pone.0156387 27227540 PMC4882051

[54] Gao J Xu K Liu H Liu G Bai M Peng C. Impact of the gut microbiota on intestinal immunity mediated by tryptophan metabolism. Front Cell Infect Microbiol. (2018) 8:13. 10.3389/fcimb.2018.00013 29468141 PMC5808205

[55] Gao K Mu CL Farzi A Zhu WY. Tryptophan metabolism: a link between the gut microbiota and brain. Adv Nutr. (2020) 11:709–723. 10.1093/advances/nmz127 31825083 PMC7231603

[56] Sepiashvili RI Balmasova IP Staurina LN. [Serotonin and its immune and physiological effects]. Ross Fiziol Zh Im I M Sechenova. (2013) 99:17–32. 23659053

[57] Alvarado DM Ciorba MA. Serotonin receptors regulate inflammatory response in experimental colitis. J Nutr. (2020) 150:1678–9. 10.1093/jn/nxaa160 32510124 PMC7330464

[58] Wang B Sun S Liu M Chen H Liu N Wu Z. Dietary L-tryptophan regulates colonic serotonin homeostasis in mice with dextran sodium sulfate-induced colitis. J Nutr. (2020) 150:1966–76. 10.1093/jn/nxaa129 32386234

[59] Belizário JE Faintuch J. Microbiome and gut dysbiosis. Exp Suppl. (2018) 109:459–76. 10.1007/978-3-319-74932-7_13 30535609

[60] Hayase E Jenq RR. Role of the intestinal microbiome and microbial-derived metabolites in immune checkpoint blockade immunotherapy of cancer. Genome Med. (2021) 13:107. 10.1186/s13073-021-00923-w 34162429 PMC8220726

[61] Ni J Wu GD Albenberg L Tomov VT. Gut microbiota and IBD: causation or correlation? Nat Rev Gastroenterol Hepatol. (2017) 14:573–584. 10.1038/nrgastro.2017.88 28743984 PMC5880536

[62] Bernstein CN Forbes JD. Gut microbiome in inflammatory bowel disease and other chronic immune-mediated inflammatory diseases. Inflamm Intest Dis. (2017) 2:116–23. 10.1159/000481401 30018962 PMC5988152

[63] Le Chatelier E Nielsen T Qin J Prifti E Hildebrand F Falony G. Richness of human gut microbiome correlates with metabolic markers. Nature. (2013) 500:541–6. 10.1038/nature12506 23985870

[64] Franzosa EA Sirota-Madi A Avila-Pacheco J Fornelos N Haiser HJ Reinker S. Gut microbiome structure and metabolic activity in inflammatory bowel disease. Nat Microbiol. (2019) 4:293–305. 10.1038/s41564-018-0306-4 30531976 PMC6342642

[65] Bangsgaard Bendtsen KM Krych L Sørensen DB Pang W Nielsen DS Josefsen K. Gut microbiota composition is correlated to grid floor induced stress and behavior in the BALB/c mouse. PLoS ONE. (2012) 7:e46231. 10.1371/journal.pone.0046231 23056268 PMC3462757

[66] Shahinozzaman M Raychaudhuri S Fan S Obanda DN. Kale attenuates inflammation and modulates gut microbial composition and function in C57BL/6J mice with diet-induced obesity. Microorganisms. (2021) 9:238. 10.3390/microorganisms9020238 33498853 PMC7911404

[67] Chen Q Wang Y Jiao F Shi C Pei M Wang L. Betaine inhibits Toll-like receptor 4 responses and restores intestinal microbiota in acute liver failure mice. Sci Rep. (2020) 10:21850. 10.1038/s41598-020-78935-6 33318565 PMC7736280

[68] Zhao Z Chen L Zhao Y Wang C Duan C Yang G. Lactobacillus plantarum NA136 ameliorates nonalcoholic fatty liver disease by modulating gut microbiota, improving intestinal barrier integrity, and attenuating inflammation. Appl Microbiol Biotechnol. (2020) 104:5273–82. 10.1007/s00253-020-10633-9 32335723

[69] Zha Z Lv Y Tang H Li T Miao Y Cheng J. An orally administered butyrate-releasing xylan derivative reduces inflammation in dextran sulphate sodium-induced murine colitis. Int J Biol Macromol. (2020) 156:1217–33. 10.1016/j.ijbiomac.2019.11.159 31759015

[70] Diling C Longkai Q Yinrui G Yadi L Xiaocui T Xiangxiang Z. CircNF1-419 improves the gut microbiome structure and function in AD-like mice. Aging. (2020) 12:260–87. 10.18632/aging.102614 31905172 PMC6977659

[71] Fu G Zhao K Chen H Wang Y Nie L Wei H. Effect of 3 lactobacilli on immunoregulation and intestinal microbiota in a β-lactoglobulin-induced allergic mouse model. J Dairy Sci. (2019) 102:1943–58. 10.3168/jds.2018-15683 30660420

[72] Singh V Yeoh BS Xiao X Kumar M Bachman M Borregaard N. Interplay between enterobactin, myeloperoxidase and lipocalin 2 regulates *E. coli* survival in the inflamed gut. Nat Commun. (2015) 6:7113. 10.1038/ncomms8113 25964185 PMC6336494

[73] Saha P Chassaing B Yeoh BS Viennois E Xiao X Kennett MJ. Ectopic expression of innate immune protein, lipocalin-2, in lactococcus lactis protects against gut and environmental stressors. Inflamm Bowel Dis. (2017) 23:1120–32. 10.1097/MIB.0000000000001134 28445245 PMC5469687

[74] Moschen AR Gerner RR Wang J Klepsch V Adolph TE Reider SJ. Lipocalin 2 protects from inflammation and tumorigenesis associated with gut microbiota alterations. Cell Host Microbe. (2016) 19:455–69. 10.1016/j.chom.2016.03.007 27078067

[75] He R Li Y Han C Lin R Qian W Hou X. L-Fucose ameliorates DSS-induced acute colitis via inhibiting macrophage M1 polarization and inhibiting NLRP3 inflammasome and NF-kB activation. Int Immunopharmacol. (2019) 73:379–88. 10.1016/j.intimp.2019.05.013 31132733

[76] Ke J Li Y Han C He R Lin R Qian W. Fucose ameliorate intestinal inflammation through modulating the crosstalk between bile acids and gut microbiota in a chronic colitis murine model. Inflamm Bowel Dis. (2020) 26:863–73. 10.1093/ibd/izaa007 32010956

