# Supplementary figures and images for: Integrated Analysis Reveals the Targets and Mechanisms in Immunosuppressive Effect of Mesalazine on Ulcerative Colitis
Source: Front Nutr. 2022 May 19;9:867692. doi: 10.3389/fnut.2022.867692 (PMC9161553; doi:10.3389/fnut.2022.867692)

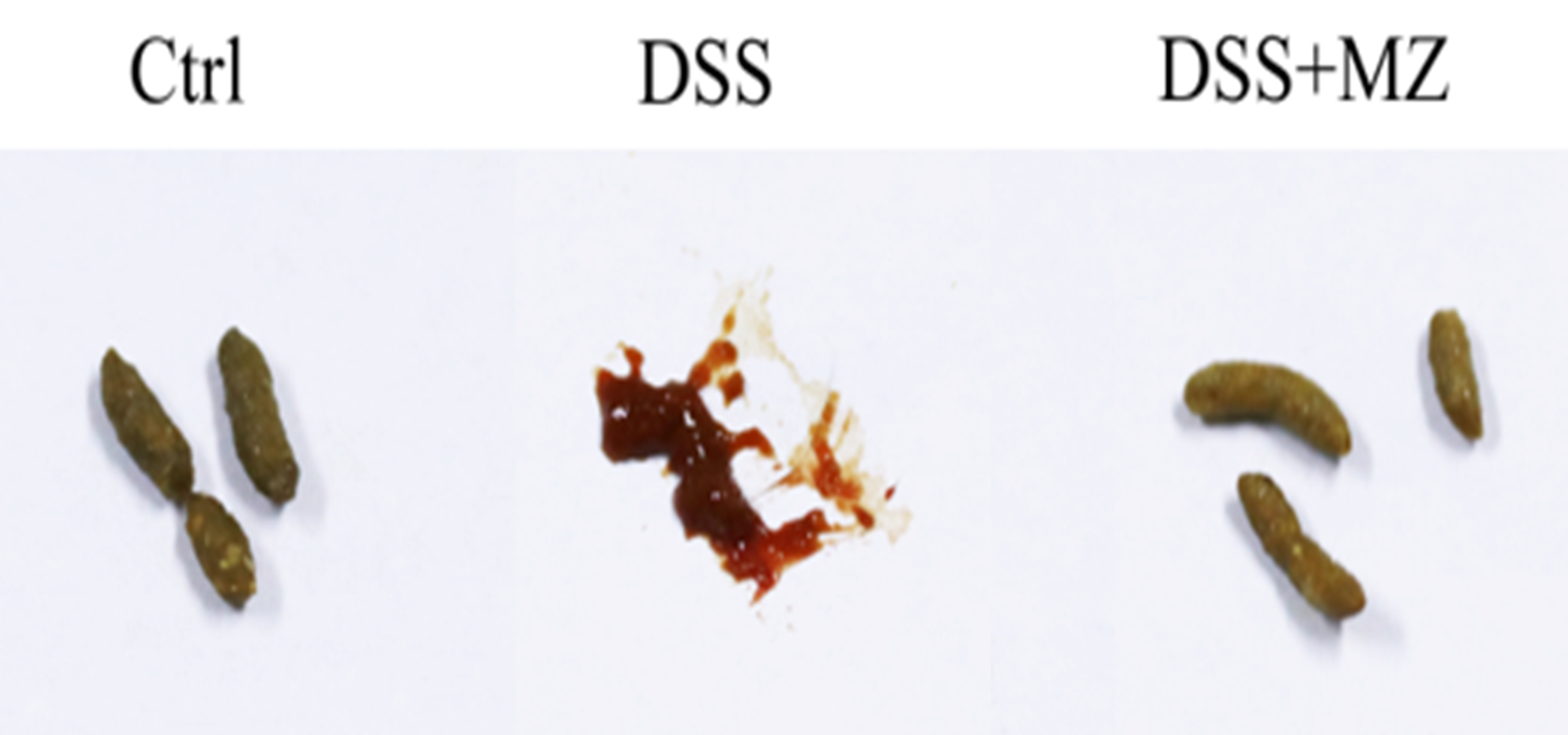

Supplement: Supplementary Figure 1 — The pictures of stool samples collected from control, DSS-induced colitis group and DSS-induced colitis + MZ group. [file Image_1.TIF]

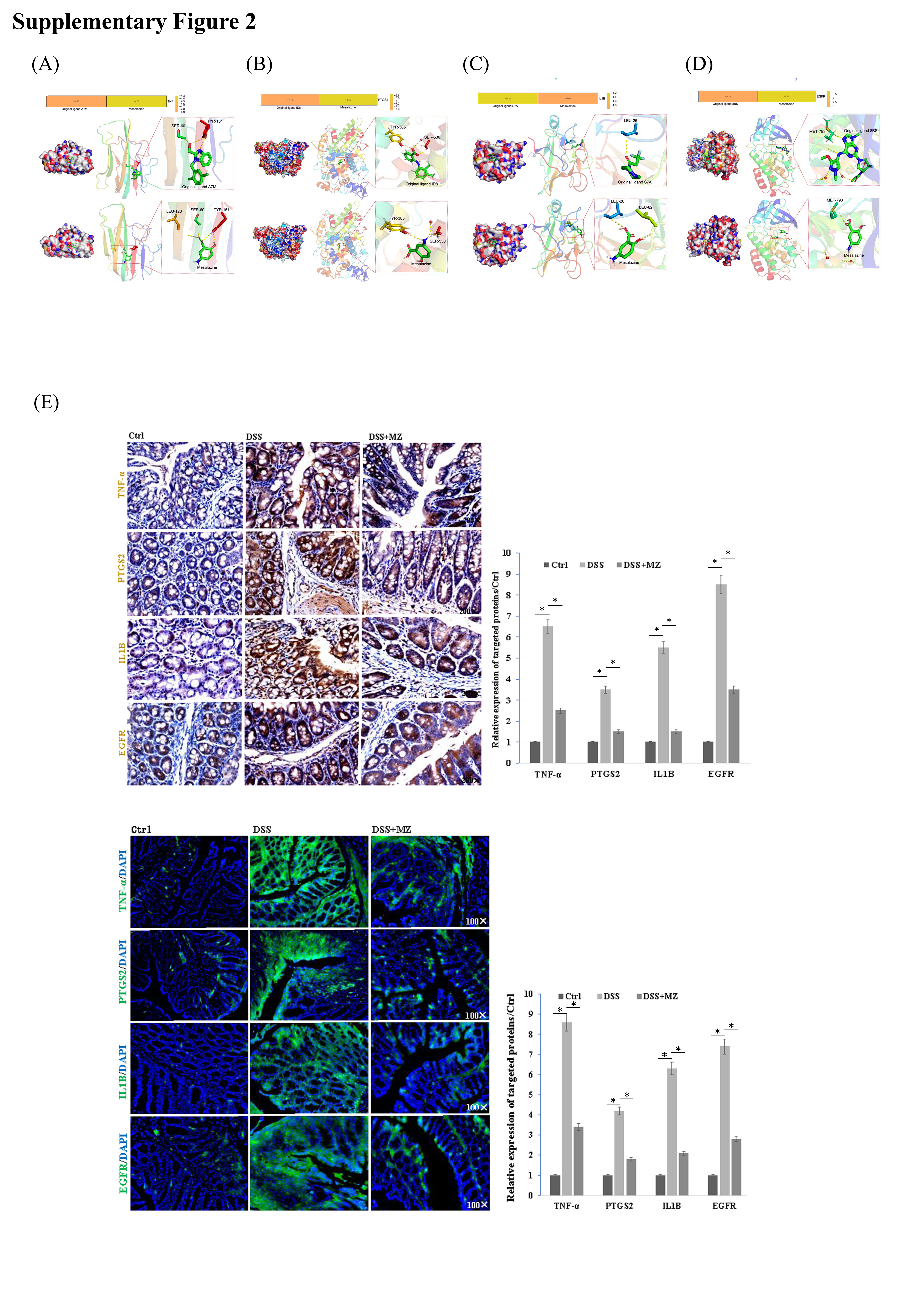

Supplement: Supplementary Figure 2 — MZ targeted relieved the elevation of TNF-α, PTGS2, IL-1β, and EGFR in DSS-induced UC model. (A) The binding of MZ with TNF. (B) The binding of MZ with PTGS2. (C) The binding of MZ with IL-1β (D) The binding of MZ with EGFR. (E) Immunostaining analysis demonstrated that MZ treatment markedly suppressed the induced expressions of TNF-α, PTGS2, IL-1β, and EGFR in colorectal of DSS-induced UC model. The experimental data were expressed as the mean ± standard deviation. Post-hoc pairwise comparisons between the groups were performed with the Dunn's test, to test statistical significance between the groups. The experimental data were expressed as the mean ± standard deviation. Significant results were determined using a cutoff of p < 0.05 and represented by asterisk. [file Image_2.tif]
